# Case report: An ectopic adrenocortical adenoma in the renal sinus

**DOI:** 10.3389/fonc.2022.934862

**Published:** 2022-07-28

**Authors:** Tianyu Liu, Renguang Lv, Xiaolin Hu, Kewei Li, Qiangqiang Ren, Yongzhen Zhang, Liwei Meng, Zhaoxu Liu, Zhaoxin Guo, Yufeng Cheng

**Affiliations:** ^1^ Department of Urology, Qilu Hospital of Shandong University, Jinan, China; ^2^ Department of Urology, Jinan Seventh People’s Hospital, Jinan, China; ^3^ Department of Endocrine, Jinan central hospital of Shandong University, Jinan, China; ^4^ Department of Urology, Zhucheng people’s Hospital Affiliated to Weifang Medical College, Weifang, China; ^5^ Department of Urology, Wucheng People’s Hospital, Dezhou, China; ^6^ The Key Laboratory of Cardiovascular Remodeling and Function Research, Chinese Ministry of Education and Chinese Ministry of Public Health, Department of Cardiology, Qilu Hospital of Shandong University, Jinan, China; ^7^ School of Nursing, Shandong University, Jinan, China; ^8^ Department of Radiation Oncology, Qilu Hospital of Shandong University, Jinan, China

**Keywords:** ectopic, nonfunctional adenomakidney, kidney, renal sinus mass, adrenocortical adenoma, laparoscopic surgery

## Abstract

**Background:**

Ectopic adrenal tissue is rare in adults, with an incidence of only about 1%. We report a rare case of ectopic adrenocortical adenoma in the left renal sinus.

**Case Preentation:**

A 57-year-old woman was admitted to the Department of Urology due to “a left kidney tumor” on physical examination. Multislice helical computed tomography (CT) showed the left kidney with an anterior lip mass near the hilum, approximately 2.3 cm × 2.2 cm in size. Preoperative renal artery CT angiography (CTA) showed no obvious abnormality. Laparoscopic resection of the left renal sinus mass was performed, and postoperative pathological findings showed ectopic adrenocortical adenoma. The tumor was a nonfunctional adenoma.

**Conclusion:**

Renal ectopic adrenal cortical adenoma is rare. Most of them are nonfunctional adenomas, which cannot be clearly diagnosed by preoperative imaging examination and can often be diagnosed by postoperative pathology.

## Introduction

Ectopic adrenal tissue is rare in adults, with an incidence of only about 1% (1). The most common sites of ectopic adrenocortical neoplasm include the celiac axis (2), broad ligament (3), testicular appendage, and spermatic cord (4). Rare ectopic adrenocortical adenomas have also been reported in the lungs (5), stomach, spinal area (6), and brain. Adrenal neoplasm adjacent to the renal sinus is very rare. We report a case of adrenal cortical adenoma of the left renal sinus.

## Case description

**Figure 1:**
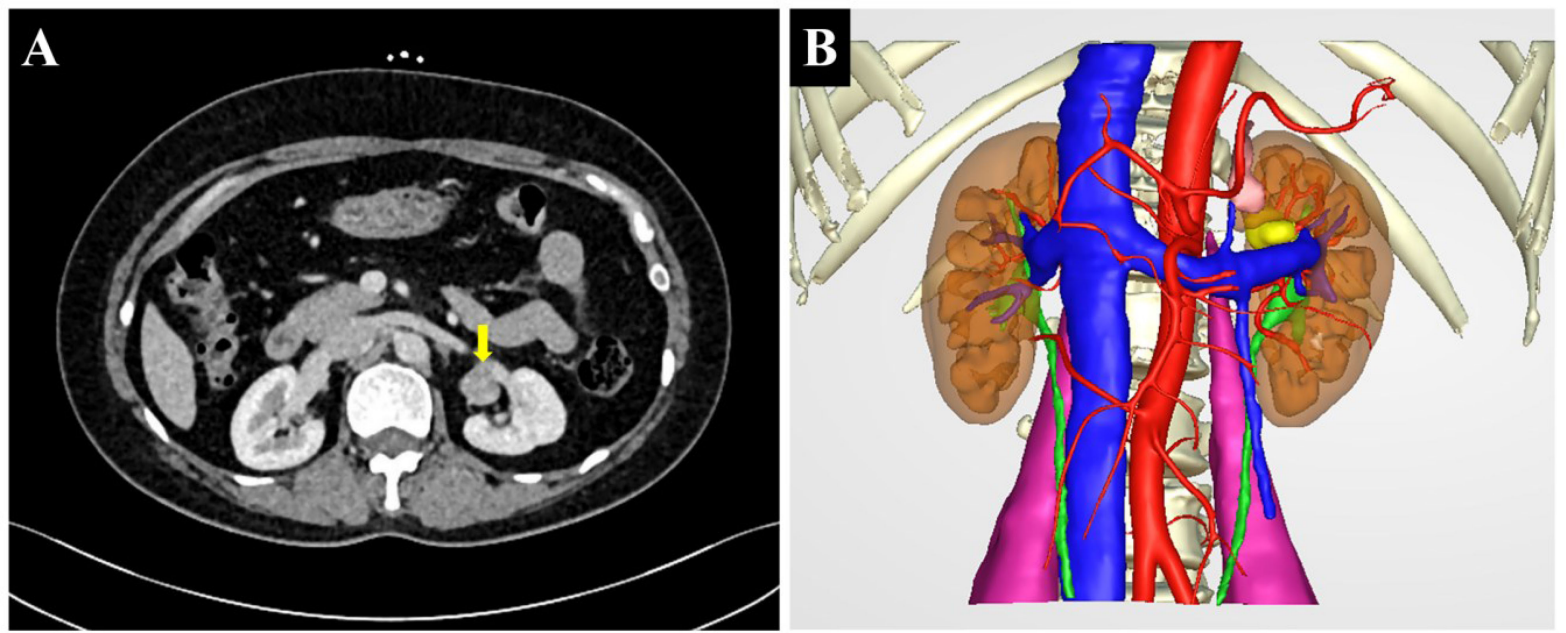
**(A)** Multislice spiral computed tomography (CT) showing a lobulated soft tissue density nodule above the hilum in the medial upper pole of the left kidney (arrow). **(B)** The preoperative three-dimensional reconstruction image showing the tumor in the renal sinus (yellow).

**Figure 2:**
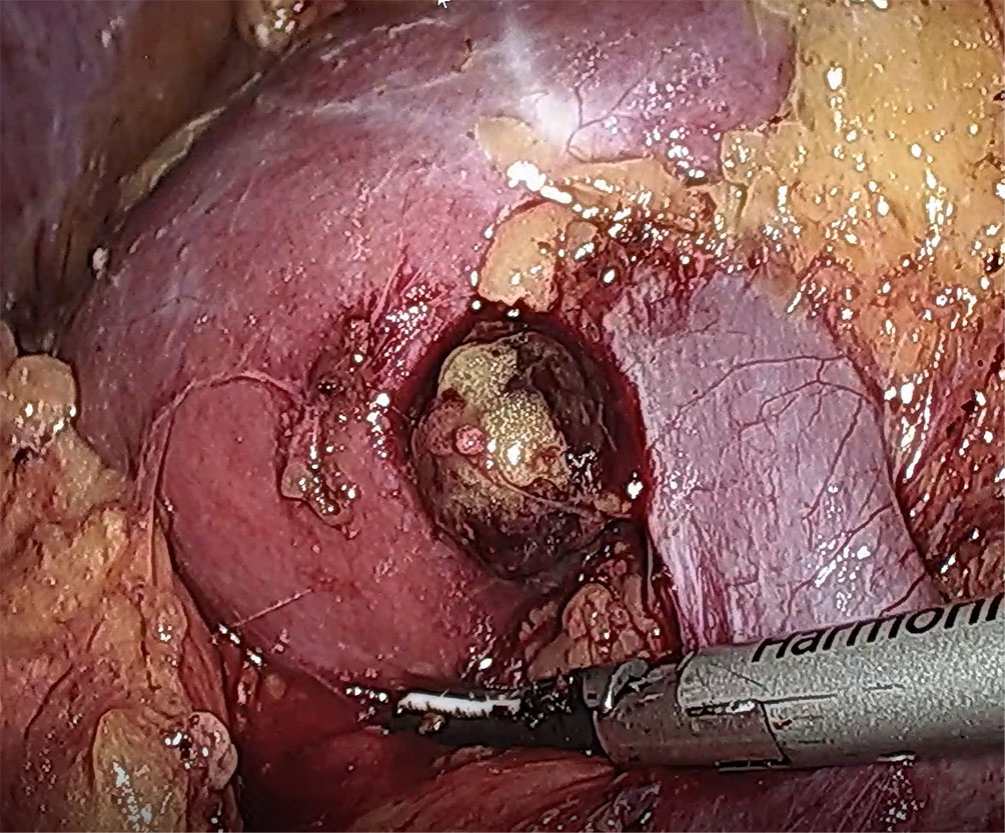
Real-time image of intraoperative ectopic adrenal tumor. The tumor was located behind the anterior lip of the left kidney, and the lower edge of the tumor was adjacent to the renal vein.

The 51-year-old female patient was admitted to the Department of Urology on 3 November 2021 after physical examination found “a left kidney tumor”. The patient did not have the common concomitant symptoms of renal tumors such as gross hematuria and lumbago. The patient’s body mass index (BMI) was 27.8 (height 168 cm, weight 78.5 kg). She was not observed to be concentrically obese, and her blood pressure (BP) was in the normal range (BP 133/84 mmHg). The physical exam was normal. No masculine features were observed. Blood routine, routine urine examination, blood sugar, blood lipid, serum electrolytes, liver function, and renal function were normal. Serum cortisol and plasma adrenocorticotropic hormone (ACTH) were not measured. MRI showed an approximately circular signal of slightly short T1 and short T2 near the renal sinus of the upper left kidney. Diffusion-weighted imaging (DWI) showed a slightly higher signal with a diameter of about 1.5 cm. Enhanced scan showed mild enhancement. Multislice spiral computed tomography (CT) demonstrates a lobulated soft tissue density nodule above the hilum in the medial upper pole of the left kidney. The density was uniform, and the size was about 2.3 cm × 2.2 cm. Moderate enhancement was observed in the arterial phase on the enhanced scan, and the CT value was slightly of lower density in the delayed phase (**Figure 1A**). CT was considered concerning for renal cell carcinoma. There were no obvious abnormalities in renal artery CTA. The clinical diagnosis was left renal sinus mass. Preoperative RENAL score was 1 + 1 + 2 + AH + 3 = 7AH. A transabdominal laparoscopic partial nephrectomy of the left kidney was planned, and accurate evaluation and preoperative planning were performed by three-dimensional (3D) visualization and reconstruction technology (**Figure 1B**). Intraoperatively, the mass was located behind the anterior lip of the left kidney. About two-fifths of the tumor volume was located in the renal parenchyma, and three-fifths protruded into the renal sinus. The lower edge of the tumor was adjacent to the renal vein (**Figure 2**). The left adrenal gland was in normal position, and no abnormalities were observed during the operation. The tumor was brittle and yellow-brown, adjacent to the renal pedicle vessels, and its base was located below the anterior lip of the kidney. The possibility of renal hamartoma was considered during the operation. Then, the tumor was enucleated. The specimen was resected and sent for routine pathological examination. The total operation duration was 1 h 35 min, and the renal pedicle vessels were blocked for 25 min. The blood loss was about 20 ml. Renal function was checked on the second day after the operation, and serum creatinine was normal. The abdominal drainage tube was removed on the third postoperative day, and the postoperative hospital stay was 4 days. Postoperative pathological findings, combined with immunohistochemistry, were consistent with ectopic adrenocortical adenoma. Three months after the operation, the patient was followed up by CT, and no residual tumor was identified.

### General features

The size of the tumor was about 3 cm × 2.2 cm × 0.8 cm, and the section was yellowish-brown.

### Microscopic characteristics

**Figure 3:**
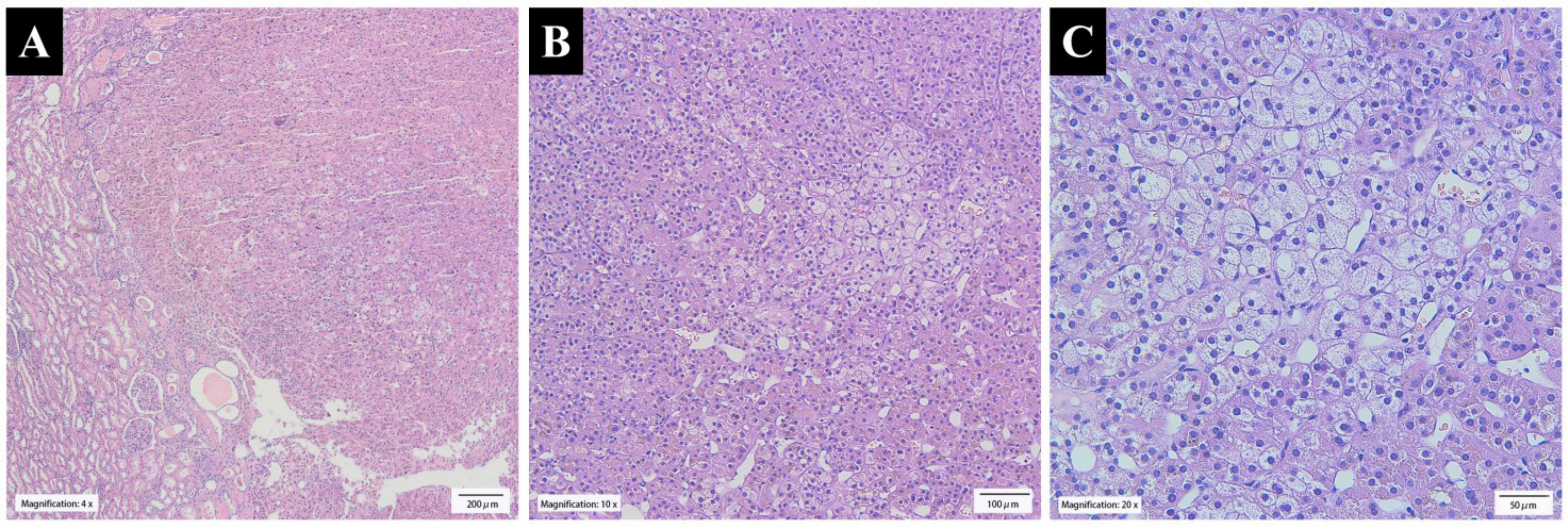
**(A)** The tumor was well circumscribed and surrounded by non-neoplastic renal parenchyma (×4 H&E stain). **(B)** The tumor growth pattern was characterized by nests, cords, and acini of adrenal cortical cells separated by abundant sinusoids and little stroma (×10 H&E stain). **(C)** The clear cells in the tumor were large and polygonal with small nuclei, and the cytoplasm was lipid-rich and vacuolated, resembling normal zona fasciculata (×20 H&E stain).

Histologically, the tumor growth pattern was characterized by nests, cords, and acini of adrenal cortical cells separated by abundant sinusoids and little stroma. Most of the tumor cells resembled normal zona fasciculata cells, with some more eosinophilic resembling normal zona glomerulosa. The tumor showed mild degenerative endocrine-type atypia, and mitotic figures were absent. The tumor was well circumscribed and surrounded by non-neoplastic renal parenchyma (**Figure 3**).

### Immunohistochemistry

**Figure 4:**
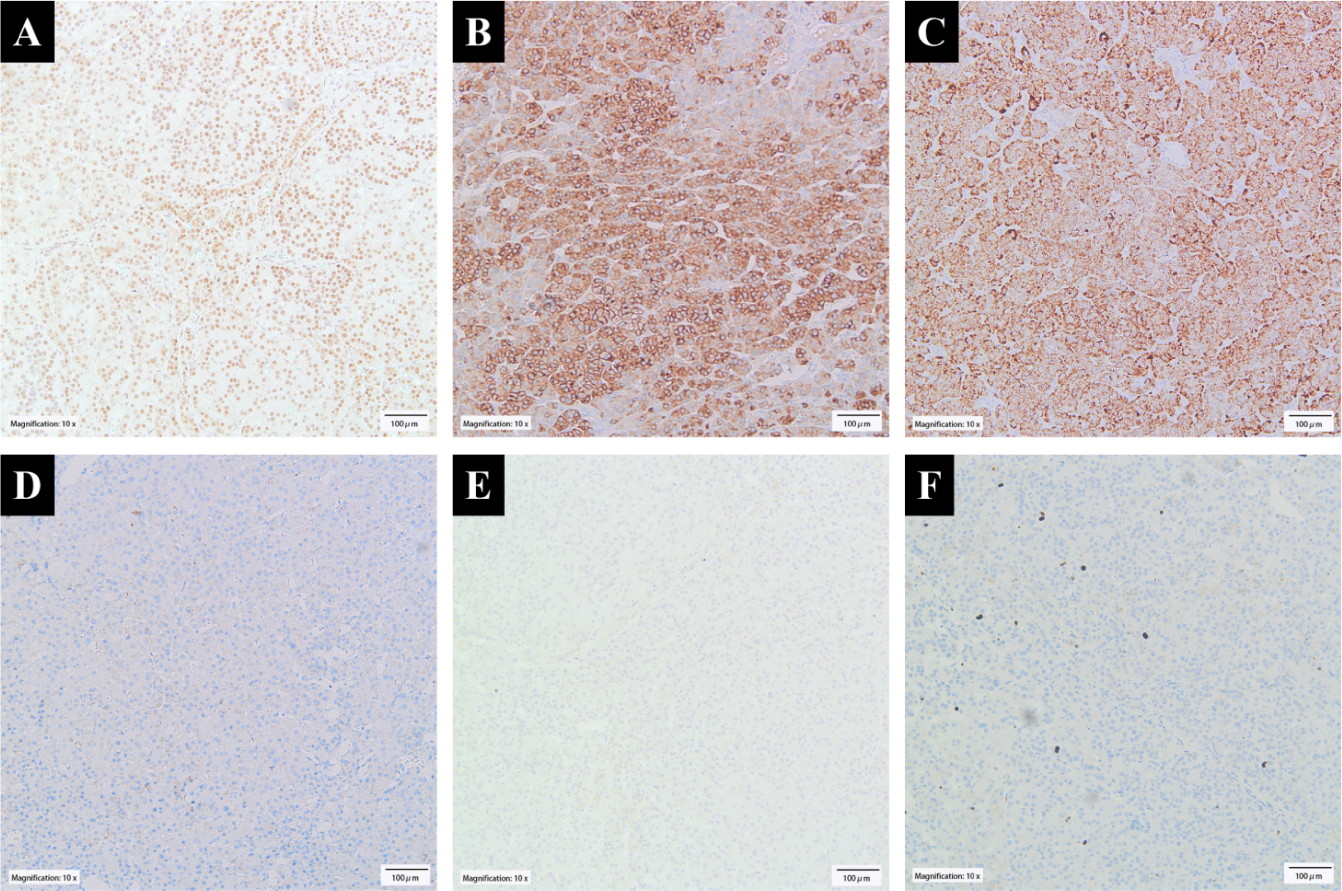
Immunohistochemical findings. **(A)** Tumor cells showed nuclear SF-1 expression. Their cytoplasm showed positive staining for **(B)** Syn and **(C)** Melan-A but negative for **(D)** CK and **(E)** CgA. **(F)** Ki-67 labeled approximately 3% of the lesional cells.

Immunohistochemically, the lesion cells were positive for SF-1, Syn, Melan-A, and HMB45 and negative for CK, CgA, EMA, CA IX, CD10, CK7, and CD117. Ki-67 labeled approximately 3% of the lesional cells (**Figure 4**).

## Discussion

The adrenal gland has a dual embryonic origin. The adrenal cortex is derived from the coelomic mesoderm of the urogenital crest and the adrenal medulla from the neural crest. During the fourth and fifth weeks of embryonic life, mesothelial cells from the angle between the root of the mesentery and the developing gonad proliferate, separate from the coelomic epithelium, and condense in the mesenchyme of the dorsal abdominal wall to form the primitive cortex (7). At week 6, a second wave of mesothelial cells surrounds the primitive cortex, which then forms the adult or final cortex. By 8 weeks, the cortical mass separates from the rest of the mesothelial tissue and is surrounded by connective tissue (8). During development, adrenal tissue breaks and fragments remain in the migration path of the urogenital tract, forming ectopic adrenal tissue (9). If this occurs after the migration of the neural crest tissue into the cortex, the ectopic adrenal tissue contains the cortex and medulla. Otherwise, only cortex exists in ectopic adrenal tissue (10). Most ectopic adrenal glands contain only cortex and very little medulla.

In general, ectopic adrenal tissue shrinks and disappears with age. So ectopic adrenal tumors are more common in children than in adults. Ectopic adrenal tissue is reported in approximately 50% of neonates and children (7) compared with only 1% of adults (1). A comprehensive review of the literature showed that accessory and heterotopic adrenal tissues were most commonly found in the area of the celiac axis (32%), followed by the broad ligament (23%), adnexa of testes (7.5%), and spermatic cord (3.8%–9.3%). Accessory adrenal tissue was found in the kidney in only 0.1%–6% of the cases reviewed, predominantly located in the subcapsular area of the upper pole (11).

The renal sinus is the medial space of the kidney surrounded by the renal parenchyma. Renal sinus tumors vary in type. Primary tumors include tumors of the renal pelvis, hemangioma, smooth muscle tumors, nerve sheath tumors, and liposarcoma. Our review of the literature found that ectopic renal adrenocortical adenomas were mostly located in the renal sinus, suggesting that ectopic adrenocortical adenomas should be considered in the differential diagnosis of renal sinus tumors.

Most reported ectopic renal adrenal cortical adenomas are nonfunctional. Ectopic cortisol-producing adrenocortical adenoma (CPA) is extremely rare (12). Functional ectopic adrenal tumors are more likely to be detected than nonfunctional adrenal tumors because of their typical clinical presentation.

This case was a nonfunctional renal ectopic adrenal adenoma. Due to the absence of abnormal laboratory studies, this case was not diagnosed before surgery. Masses that are adjacent to the renal sinus can be difficult to precisely localize and diagnose preoperatively. Biopsy may be helpful, but surgical resection of the lesions may still be necessary for a definitive pathological diagnosis.

The immunoprofile (SF-1 +, Syn +, Melan-A +, HMB45 +, CK -, CgA -, EMA -, CA IX -, CD10 -, CK7 -, and CD117 -) supported the diagnosis of adrenal cortical adenoma. The cells of adrenal cortical adenomas are immunoreactive for several proteins, including SF-1, Syn, Melan-A, and HMB45, as shown in this case. CK expression is usually weak or absent. In contrast to medullary tumors, CgA is negative. Lack of EMA, CA IX, and CD10 expression excludes clear cell renal cell carcinoma. Renal oncocytic tumors can also be excluded by SF-1 positivity. The Ki-67 proliferation is quite low (about 3%), consistent with lack of mitosis and necrosis and further arguing against the diagnosis of adrenal cortical carcinoma.

Management of ectopic adrenal tumors includes conservative treatment and surgical resection (open or minimally invasive). Some ectopic adrenal tumors are easy to find because of endocrine function and clinical symptoms. In such cases, early treatment should be given. If the tumor is small and has no endocrine function, conservative observation can be selected. The vast majority of adrenal neoplasms are benign, but preoperatively identifying the mass as ectopic adrenal is a challenge.

Malignant tumor could not be ruled out before surgery in this case. Renal angiomyolipoma was a consideration intraoperatively, but intraoperative pathological diagnosis was not performed due to surgical considerations. Intraoperatively, the tumor did not invade surrounding tissues and was enucleated. Pathologically, there was no evidence of malignancy.

## Conclusion

In this case, the patient was diagnosed with a renal tumor preoperatively. Due to the absence of endocrine dysfunction, the tumor was not accurately diagnosed before surgery. Postoperative pathologic diagnosis was adrenal cortical adenoma. Since the patient had no history of adrenal surgery, the combined findings were those of an ectopic adrenal cortical adenoma, presumably a result of abnormal migration of mesothelial cells during embryology. In addition, combined with literature reports, we found that ectopic adrenal tumors in the kidney were mostly located in the soft tissue of the renal sinus. Therefore, it is suggested that ectopic adrenocortical adenoma should be considered in the differential diagnosis of renal sinus masses. CT can be used to observe whether adrenal atrophied, and serum cortisol and plasma ACTH levels can be measured by laboratory tests, or a preoperative biopsy of the tumor, if possible, should be performed to confirm the diagnosis of ectopic adrenocortical adenoma, so as to provide better support for surgical decisions.

## Data availability statement

The original contributions presented in the study are included in the article/**Supplementary Material**. Further inquiries can be directed to the corresponding author/s.

## Ethics statement

Written informed consent was obtained from the individual(s) for the publication of any potentially identifiable images or data included in this article.

## Author contributions

TL and RL provided patient information and wrote the manuscript. XH conceived the manuscript. KL and QR collected the data. YZ and LM prepared histopathological examination and illustrations. ZL consulted the treatment plan. ZG reviewed the topic presentation, structure of the manuscript, illustrations and photographs. YC obtained resources, reviewed and edited the writing. All authors contributed to the article and approved the submitted version.

## Conflict of interest

The authors declare that the research was conducted in the absence of any commercial or financial relationships that could be construed as a potential conflict of interest.

## Publisher’s note

All claims expressed in this article are solely those of the authors and do not necessarily represent those of their affiliated organizations, or those of the publisher, the editors and the reviewers. Any product that may be evaluated in this article, or claim that may be made by its manufacturer, is not guaranteed or endorsed by the publisher.
