# Supplementary figures and images for: Case report: An ectopic adrenocortical adenoma in the renal sinus
Source: Front Oncol. 2022 Jul 28;12:934862. doi: 10.3389/fonc.2022.934862 (PMC9366061; doi:10.3389/fonc.2022.934862)

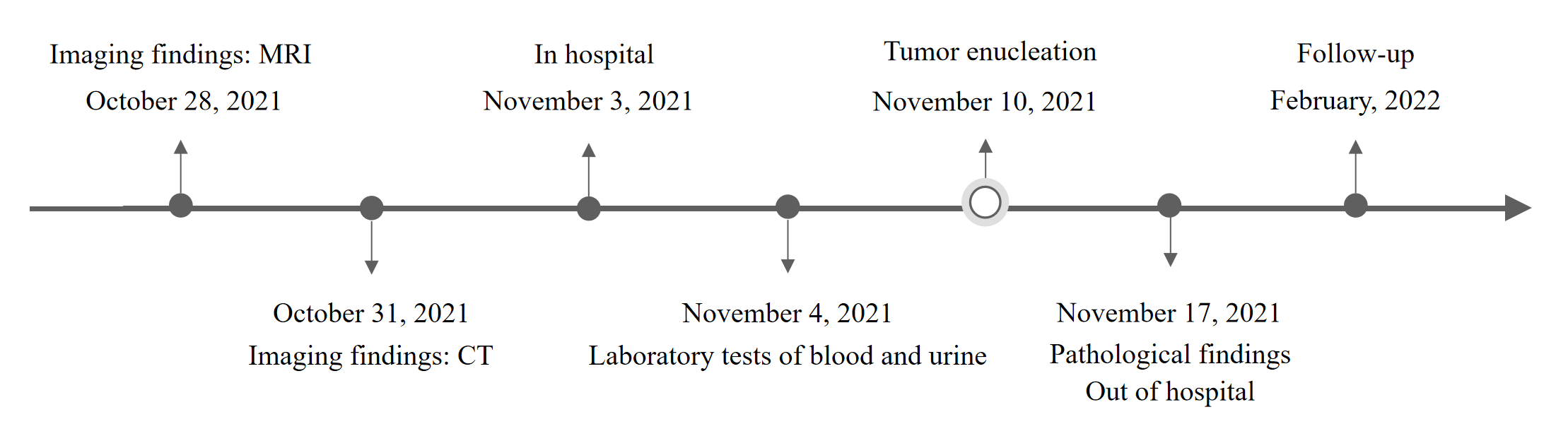

Supplement: Supplementary file 1 [file Image_1.tif]
